# Protocol to purify fatty acid metabolites with two approaches for quantification using liquid chromatography-mass spectrometry

**DOI:** 10.1016/j.xpro.2026.104459

**Published:** 2026-03-22

**Authors:** Tomomi Hashidate-Yoshida, Masaki Yamada, Katsuyuki Nagata, Yasuyuki Kihara, Yoshihiro Kita, Hideo Shindou

**Affiliations:** 1 Department of Lipid Life Science, National Institute of Global Health and Medicine, Japan Institute Health Security (JIHS), Shinjuku-ku, Tokyo 162-8655, Japan; 2 Shimadzu Corporation, Nakagyo-ku, Kyoto 604-8511, Japan; 3 Department of Molecular Biology, The Jikei University School of Medicine, Minato-ku, Tokyo 105-8461, Japan; 4 Center for Neurologic Diseases, Sanford Burnham Prebys Medical Discovery Institute, La Jolla, CA 92037, USA; 5 Life Sciences Core Facility, Graduate School of Medicine, The University of Tokyo, Bunkyo-ku, Tokyo 113-0033, Japan; 6 Department of Medical Lipid Science, Graduate School of Medicine, The University of Tokyo, Bunkyo-ku, Tokyo 113-0033, Japan

**Keywords:** Cell Membrane, Health Sciences, Metabolism, Signal Transduction

## Abstract

Fatty acid metabolites, such as eicosanoids and docosanoids, play important biological roles and are strictly regulated by diverse enzymes. Here, we present two approaches for purifying their metabolites, including the phospholipid mediator platelet-activating factor (PAF), for quantification using liquid chromatography-mass spectrometry (LC-MS). We describe steps for tissue collection, lipid extraction, and lipid mediator purification. We then detail procedures for data analysis. This protocol allows the selection of a suitable technique for analyzing target molecules.

For complete details on the use and execution of this protocol, please refer to Yamamoto et al.^1^

## Highlights

• Instructions for the detection of fatty acid metabolites
• Steps for the purification and enrichment of fatty acid metabolites
• Selection of the purification procedure for the target metabolites

## Abstract (editor highlights)

Publisher’s note: Undertaking any experimental protocol requires adherence to local institutional guidelines for laboratory safety and ethics.

## Graphical abstract

**Figure:**
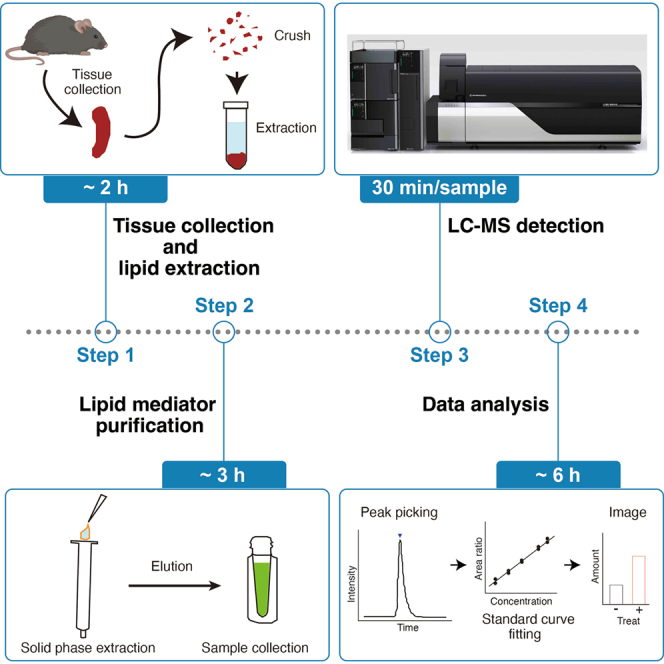

## Before you begin

Fatty acids are released expeditiously in response to extracellular stimuli, and diverse metabolites are generated through several pathways. The generation of fatty acid metabolites depends on the tissues and the time course after stimulation. Another group^2^ and we^3^^,^^4^ have independently reported the lipidomic procedures to analyze fatty acids and their metabolites using a liquid chromatography–mass spectrometry (LC–MS) and a gas chromatography.^5^ In the methods of Yamada *et al.*,^4^ 141 fatty acids and their metabolites were identified. Turtoi *et al.* also reported the method to measure 163 polyunsaturated fatty acids (PUFA)s and oxylipins.^2^ In this protocol, 199 molecules of fatty acids and their metabolites were measured. In addition to the previous methods,^2^^,^^4^ DHA metabolites (Hydroxy-DHAs, EpDPA, DiHDHA, and Resolvin D3, D4, D5) and EPA metabolites (LTB_5_, 14,15-EpETE, 11-HEPE, and 9-HEPE) were added. Moreover, we proposed two purification methods, “Purification Protocol 1: Conventional protocol” and “Purification Protocol 2: Improved protocol”, for fatty acid metabolites prior to LC–MS analysis. A suitable method can be selected based on the type of the fatty acid metabolites.

1. Preparation of internal standards (ISTD) and standards (STD)
  a. Prepare the 20× ISTD using 24 deuterium-labeled compounds to a final concentration of 1, 2, 4, 10 or 40 ng/μl (Table S1).
    ***Note:*** The 20× ISTD is stable for 1 year at −80°C.
  b. Prepare the STD group1, 2, 3, 4 and 5 to a final concentration of 1000 pg/μL STD group1, 2, 3, 4 and 10000 pg/μL STD group5 (Table S1).
  c. Dilute 20× ISTD with methanol to 1× ISTD.
  d. Prepare the dilution buffer.
  e. Dilute STD group1, 2, 3, 4 and 5 10-fold with methanol or dilution buffer.
    **Table:** 1× ISTD

Reagent	Final concentration	Amount
20× ISTD	1× ISTD, 40 ng/μL-EPA-d_5_, DHA-d_5_, AA-d_8_, and LA-d_4_, 4 ng/μL -AEA-d_4_, 2 ng/uL -lyso-PAF-d_4_, 1 ng/uL -OEA-d_4_, 10 ng/uL -the other 17 compounds	5 μL
MeOH	N/A	95 μL
**Total**	**N/A**	**100** μL
    **Table:** Dilution buffer (S0: 0.1× ISTD)

Reagent	Final concentration	Amount
1× ISTD	0.1 × ISTD	50 μL
MeOH	N/A	450 μL
**Total**	**N/A**	**500 μL**
    **Table:** S100 STD

Reagent	Final concentration	Amount
1000 pg/μL STD group1	100 pg/μL STD group1	10 μL
1000 pg/μL STD group2	100 pg/μL STD group2	10 μL
1000 pg/μL STD group3	100 pg/μL STD group3	10 μL
1000 pg/μL STD group4	100 pg/μL STD group4	10 μL
10000 pg/μL STD group5	1000 pg/μL STD group5	10 μL
1× ISTD	0.1 × ISTD	10 μL
MeOH	N/A	40 μL
**Total**	**N/A**	**100 μL**
    **Table:** S10 STD

Reagent	Final concentration	Amount
S100	10 pg/μL STD group1, 2, 3, 4 100 pg/μL STD group5 0.1 × ISTD	10 μL
Dilution buffer	0.1 × ISTD	90 μL
**Total**	**N/A**	**100 μL**
    **Table:** S1 STD

Reagent	Final concentration	Amount
S10	1 pg/μL STD group1, 2, 3, 4 10 pg/μL STD group5 0.1 × ISTD	10 μL
Dilution buffer	0.1 × ISTD	90 μL
**Total**	**N/A**	**100 μL**
    **Table:** S0.1 STD

Reagent	Final concentration	Amount
S10	0.1 pg/μL STD group1, 2, 3, 4 1 pg/μL STD group5 0.1 × ISTD	10 μL
Dilution buffer	0.1 × ISTD	90 μL
**Total**	**N/A**	**100 μL**
    **Table:** S0.01 STD

Reagent	Final concentration	Amount
S10	0.01 pg/μL STD group1, 2, 3, 4 0.1 pg/μL STD group5 0.1 × ISTD	10 μL
Dilution buffer	0.1 × ISTD	90 μL
**Total**	**N/A**	**100 μL**
2. LC–MS setup for fatty acids and their metabolites.
  a. Prepare the solvent A and B (100% acetonitrile).
  b. Start the LC-MS.

***Note:*** See Table 1, 2, 3, 4, and 5 for measurement conditions. LC-Nexera 40 Series UHPLC/HPLC System (Shimadzu). MS- a triple quadrupole mass spectrometer LCMS-8060 (Shimadzu)^4^ method- modified “lipid mediator ver.3” (Shimadzu).^4^

**Table:** Solvent A for LC-MS

Reagent	Final concentration	Amount
Formic acid (99.5%)	0.1%	1 mL
Water	N/A	999 mL
**Total**	**N/A**	**1000 mL**

**Table 1:** LC conditions

Column temperature	40°C
Injection volume	5.0 μL
Flow rate	0.4 mL/min

**Table 2:** LC gradient conditions

Time (min)	Gradient B (%)
0	10
5	25
10	35
20	75
20.1	95
28	95
28.1	10
30	10

**Table 3:** MS parameter settings

Scan type	MRM
Polarity	Negative (196 compounds)/Positive (27 compounds)
Ion source Nebulizer gas Drying gas CID gas Interface temperature Heat block temperature Desolvation line temperature	Heated Electrospray Ionization (ESI) nitrogen gas: 3 L/min nitrogen gas: 10 L/min Argon gas 300°C 400°C 250°C

**Table 4:** MS polarity and compound’s retention time

Name	Polarity	Ret. Time (min)
LTD_4_-d5	Pos	12.1
LTC_4_-d5	Pos	12.6
LTE_4_-d5	Pos	12.8
Lyso-PAF-d4	Pos	16.5
AEA-d4	Pos	18.2
OEA-d4	Pos	19.2
tetranor-PGEM-d6	Neg	3.2
6-keto-PGF_1a_-d4	Neg	7.8
TXB_2_-d4	Neg	9.5
PGF_2a_-d9	Neg	10.3
PGE_2_-d4	Neg	10.7
PGD_2_-d4	Neg	11.1
PGA_2_-d4	Neg	13.1
LTB_4_ d4	Neg	14.1
14,15-DiHET-d11	Neg	15.0
15-HETE-d8	Neg	16.9
12-HETE-d8	Neg	17.2
5-HETE-d8	Neg	17.3
PAF-d4	Neg	17.5
11,12-EET-d11	Neg	18.2
EPA-d5	Neg	19.4
DHA-d5	Neg	20.2
AA-d8	Neg	20.3
LA-d4	Neg	21.5
14,15-LTC_4_	Pos	11.7
14,15-LTE_4_	Pos	12.2
LTB_4_-EA	Pos	12.6
LTC_4_	Pos	12.6
11-trans-LTC_4_	Pos	12.6
LTD_4_	Pos	12.2
LTE_4_	Pos	13.1
LTF_4_	Pos	12.9
11-trans-LTD_4_	Pos	12.4
11-trans-LTE_4_	Pos	13.0
8,12-iso-iPF2a-VI-1,5-lactone	Pos	13.8
14,15-EET-EA	Pos	15.8
11,12-EET-EA	Pos	16.2
8,9-EET-EA	Pos	16.3
5,6-EET-EA	Pos	16.5
Lyso-PAF	Pos	16.5
PC 16:0_2:0	Pos	17.2
5,6-DHET-lactone	Pos	17.7
AEA	Pos	18.3
15-KEDE	Pos	18.6
OEA	Pos	19.2
tetranor-PGFM	Neg	2.8
tetranor-PGEM	Neg	3.2
tetranor-PGDM	Neg	3.4
tetranor-PGJM	Neg	4.6
tetranor-PGAM	Neg	4.8
20-hydroxy-PGF2a or 19-hydroxy-PGF_2a_	Neg	5.6
20-hydroxy-PGE_2_	Neg	5.7
18-carboxy-dinor-LTB_4_	Neg	6.2
delta17-6-keto-PGF_1a_	Neg	6.7
13,14-dihydro-15-keto-tetranor-PGF_1b_	Neg	6.7
2,3-dinor-TXB_1_	Neg	7.1
2,3-dinor-8-iso-PGF_2a_	Neg	7.3
2,3-dinor-TXB_2_	Neg	7.5
13,14-dihydro-15-keto-tetranor-PGF_1a_	Neg	7.6
2,3-dinor-11b-PGF_2a_	Neg	7.7
6-keto-PGF_1a_	Neg	7.8
13,14-dihydro-15-keto-tetranor-PGD_2_	Neg	8.0
Resolvin E1	Neg	8.1
20-carboxy-LTB_4_	Neg	8.2
PGF2a-EA	Neg	8.2
6-keto-PGE_1_	Neg	8.2
PGE2-EA	Neg	8.3
8-iso-PGF_3a_	Neg	8.3
TXB_3_	Neg	8.4
20-hydroxy-LTB_4_	Neg	8.5
PGE1-EA	Neg	8.6
11-dehydro-2,3-dinor-TXB_2_	Neg	8.5
13,14-dihydro-15-keto-tetranor-PGE_2_	Neg	8.7
2,3-dinor-PGE_1_	Neg	8.7
PGD2-EA	Neg	8.8
6,15-diketo-13,14-dihydro-PGF_1a_	Neg	9.0
PGF_3a_	Neg	9.1
iPF2a-IV	Neg	9.2
8-iso-15(R)-PGF_2a_	Neg	9.4
TXB_1_	Neg	9.4
TXB_2_	Neg	9.5
8-iso-PGF_2a_	Neg	9.5
11-dehydro-TXB_3_	Neg	9.5
PGE_3_	Neg	9.6
8-iso-PGF_1a_	Neg	9.6
11b-PGF_2a_	Neg	9.7
5-iPF2a-VI	Neg	9.9
PGD_3_	Neg	9.9
8-iso-15-keto-PGF_2a_	Neg	10.0
PGF_2a_	Neg	10.3
PGF_1a_	Neg	10.5
8-iso-13,14-dihydro-15-keto-PGF_2a_	Neg	10.5
LXA5	Neg	10.5
8-iso-PGE_2_	Neg	10.6
PGE_2_	Neg	10.8
11-dehydro-TXB_2_	Neg	10.8
8-iso-PGE_1_	Neg	10.9
15-keto-PGF_2a_	Neg	10.9
11b-PGE_2_	Neg	10.9
Resolvin D3	Neg	11.0
5S,14R-LXB4	Neg	11.1
PGK_2_	Neg	11.1
PGE_1_	Neg	11.2
PGD_2_	Neg	11.2
PGD_1_	Neg	11.3
15-keto-PGF_1a_	Neg	11.3
11b-13,14-dihydro-15-keto-PGF_2a_	Neg	11.3
15-keto-PGE_2_	Neg	11.3
Resolvin D_2_	Neg	11.4
13,14-dihydro-PGF_1a_	Neg	11.6
13,14-dihydro-PGE_1_	Neg	11.7
13,14-dihydro-15-keto-PGF_2a_	Neg	11.8
5S,6R-LXA4	Neg	11.9
13,14-dihydro-15-keto-PGE_2_	Neg	12.0
Resolvin D1	Neg	12.0
5S,6S-LXA4	Neg	12.1
1a,1b-dihomo-PGF_2a_	Neg	12.4
13,14-dihydro-15-keto-PGD_2_	Neg	12.6
Resolvin D4	Neg	12.8
8-iso-PGA_2_	Neg	12.9
13,14-dihydro-15-keto-PGD_1_	Neg	13.0
8-iso-PGA_1_	Neg	13.1
PGA_2_	Neg	13.1
LTB_5_	Neg	13.2
PGJ_2_	Neg	13.2
PGB_2_	Neg	13.2
PGA_1_	Neg	13.4
8,15-DiHETE	Neg	13.9
17,18-DiHETE	Neg	14.0
6-trans-LTB_4_	Neg	14.0
5,15-DiHETE	Neg	14.0
13,14-dihydro-15-keto-PGA_2_	Neg	14.2
Maresin1	Neg	14.1
LTB_4_	Neg	14.2
10,17-DiHDHA	Neg	14.2
13,14-dihydro-15-keto PGJ_2_	Neg	14.2
Resolvin D5	Neg	14.2
14,15-DiHETE	Neg	14.3
7,17-hydroxy-DPA	Neg	14.5
12,13-DiHOME	Neg	14.5
9,10-DiHOME	Neg	14.7
12-keto-LTB_4_	Neg	14.8
5,6-DiHETE	Neg	14.9
tetranor-12-HETE	Neg	14.8
N-acetyl-LTE_4_	Neg	15.0
LTB_3_	Neg	15.0
19,20-DiHDPA	Neg	15.1
14,15-DHET	Neg	15.1
12-HHT	Neg	15.2
11,12-DHET	Neg	15.4
8,9-DHET	Neg	15.6
20-carboxy-AA	Neg	15.7
9-HOTrE	Neg	15.7
5,6-DHET	Neg	15.8
13-HOTrE	Neg	15.9
18-HEPE	Neg	15.9
19-HETE	Neg	16.0
15-deoxy-delta-12,14-PGJ_2_	Neg	16.0
20-HETE	Neg	16.1
15-HEPE	Neg	16.2
11-HEPE	Neg	16.3
18-HETE	Neg	16.3
8-HEPE	Neg	16.3
13-HpOTrE	Neg	16.3
9-HEPE	Neg	16.4
17-HETE	Neg	16.4
12-HEPE	Neg	16.4
16-HETE	Neg	16.5
5-HEPE	Neg	16.5
15-HpEPE	Neg	16.6
13-HODE	Neg	16.6
9-HODE	Neg	16.6
12-HpEPE	Neg	16.7
20-HDHA	Neg	16.8
5-HpEPE	Neg	16.9
15-HETE	Neg	17.0
17,18-EpETE	Neg	17.0
9-HpODE	Neg	17.0
13-KODE	Neg	17.0
13-HpODE	Neg	17.0
16-HDHA	Neg	17.1
17-HDHA	Neg	17.1
9-KODE	Neg	17.2
11-HETE	Neg	17.2
13-HDHA	Neg	17.2
10-HDHA	Neg	17.2
8-HETE	Neg	17.2
14-HDHA	Neg	17.3
12-HETE	Neg	17.3
15-KETE	Neg	17.3
15-HpETE	Neg	17.3
14,15-EpETE	Neg	17.3
11-HDHA	Neg	17.3
7-HDHA	Neg	17.3
9-HETE	Neg	17.3
8-HDHA	Neg	17.4
5-HETE	Neg	17.4
17-HpDHA	Neg	17.4
15-HETrE	Neg	17.5
8-HETrE	Neg	17.5
12-HpETE	Neg	17.5
PAF	Neg	17.6
12-KETE	Neg	17.6
4-HDHA	Neg	17.7
5-HpETE	Neg	17.8
12,13-EpOME	Neg	17.8
9,10-EpOME	Neg	17.9
19,20-EpDPA	Neg	17.9
14,15-EET	Neg	18.0
5-KETE	Neg	18.1
Azelaoyl-PAF	Neg	18.2
11-HEDE	Neg	18.2
16,17-EpDPA	Neg	18.2
15-HEDE	Neg	18.2
11,12-EET	Neg	18.3
5-HETrE	Neg	18.4
8,9-EET	Neg	18.3
5,6-EET	Neg	18.4
PAF_18:0	Neg	19.1
EPA	Neg	19.5
DHA	Neg	20.2
AA	Neg	20.4
LA	Neg	20.4

**Table 5:** MRM settings of compounds not included in lipid mediator ver.3

Name	Transition Q1 (m/z)	Transition Q3 (m/z)	Collision (V)	Dwell time
LTE_4_-d5	445.3	194.1	15.0	6
Lyso-PAF-d4	486.3	104.2	25.0	6
AEA-d4	352.2	66.1	15.0	6
PGF_2a_-d9	362.2	193.1	26.0	6
EPA-d5	306.2	262.2	10.0	6
DHA-d5	333.2	288.2	11.0	6
LA-d4	283.2	283.2	8.0	6
PC 16:0_2:0	538.4	184.1	29.0	6
PAF_18:0	596.4	59.1	31.0	6
LA	279.2	279.2	8.0	6

### Innovation

We established two purification methods for fatty acid metabolites prior to LC–MS analysis: Purification Protocol 1 (conventional protocol) and Purification Protocol 2 (improved protocol). Moreover, the number of target metabolites was expanded to 199 species compared with our previous method.

### Institutional permissions (if applicable)

Spleen, brain, and lung tissues were collected from C57BL/6 mice (CLEA Japan). All animal experiments were approved by and performed in accordance with the guidelines of the Animal Research Committee of the Japan Institute for Health Security (JIHS) and conducted in accordance with its guidelines.

## Key resources table

**Table:** 

REAGENT or RESOURCE	SOURCE	IDENTIFIER
**Chemicals, peptides, and recombinant proteins**		

Acetonitrile (LC/MS grade)	Wako	Cat#018-19853; CAS: 75-05-8
Methanol (LC/MS grade)	Wako	Cat#134-14523; CAS: 67-56-1
Formic Acid (abt. 99%)	Wako	Cat#063-04192; CAS: 64-18-6
2-Propanol (LC/MS grade)	Wako	Cat#168-25531; CAS: 67-63-0
Ethanol (99.5)	Wako	Cat#057-00456; CAS: 64-17-5
Petroleum Ether	Wako	Cat#161-00805; CAS: 8032-32-4
Liquid nitrogen	Any	N/A

**Experimental models: Organisms/strains**		

Mouse; C57BL/6J	N/A	N/A

**Software and algorithms**		

LabSolutions Insight	Shimadzu	-

**Other**		

AUTO MILL	Tokken	TK-AM7-48
Rotary evaporator	TAITEC	VC-96R VC-96W
Freeze Trap	TAITEC	VA-500R
Fume hood	DALTON	DFB13-AA18-LA40
Manifold	Waters	WAT200677
Vacuum pump	ULVAC	MDA-015
Rotator	TAITEC	RT-50
Vortex	Scientific Industries	SI-A286
Milli-Q water maker	Merck	Integral 5
Kinetex 2.6 μm C8 100 Å, LC Column 150 × 2.1 mm	Phenomenex	Cat#00F-4497-AN
Liquid chromatograph-mass spectrometry	Shimadzu	LCMS-8060
2.0 mL tube (stainless inner lid)	Bio Medical Science	Cat#MT020-01HS
Crusher	Tokken	Cat#SK-100-D100
Glass Test Tube Phi 16 × 125 mm	KIMBLE	Cat#73500-16125
12 mL Test Tube PP	nerbe plus	Cat#02-222-0000
Plastic vial	GL Sciences	Cat#1030-14110
Oasis HLB 1 cc Vac Cartridge, 10 mg Sorbent per Cartridge, 30 μm	Waters	Cat#186000383
Capillary & Piston CP10	Gilson	Cat#F148312
Capillary & Piston CP100	Gilson	Cat#F148314
Capillary & Piston CP1000	Gilson	Cat#F148560
Microman E M10E	Gilson	Cat#FD10001
Microman E M100E	Gilson	Cat#FD10004
Microman E M1000E	Gilson	Cat#FD10006

## Step-by-step method details

In this section, we first describe the extraction and purification of fatty acids and their metabolites from tissues. We then outline their detection using LC–MS, followed by procedures for data analysis.

### Tissue crush

**Timing: 1 h**

**CRITICAL:** Operators should equip safety goggles, gloves, and a lab coat, when organic solvents and liquid nitrogen are used. All experiments using organic solvents should be done in a draft chamber.

This step describes a procedure for tissue disruption using an Automill.

**Figure 1:**
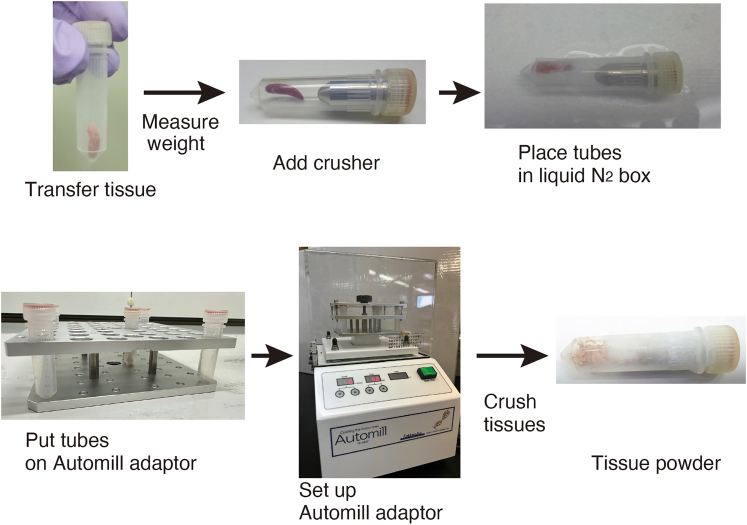
A workflow for tissue crush

The overview for tissue disruption is summarized in Figure 1.

1. Transfer 10–100 mg of tissue to a specific tube, measure the tissue weight, and insert the metal crusher into the tube (Figure 1).
2. Disrupt the issue into a fine powder using an Automill (Tokken Inc.) (Figure 1).

### Extraction of fatty acids and their metabolites

**Timing: 2 h**

This step describes the procedure for the extraction of fatty acids and their metabolites from powdered tissues.

3. Add 0.8 mL methanol to the triturated sample, followed by 0.01 mL of 1× ISTD in methanol.

***Note:*** If the tube cracks at the cap or bottom, transfer the sample to a new tube.

4. Mix the sample on a rotator for 1 h at 4°C.
5. Remove the crusher from the tube using forceps.
6. Centrifuge at 20,000 × *g* for 10 min at 4°C.
7. Collect 0.65 mL of supernatant.

### Purification and enrichment of fatty acid metabolites

**Timing: 2 h**

**Figure 2:**
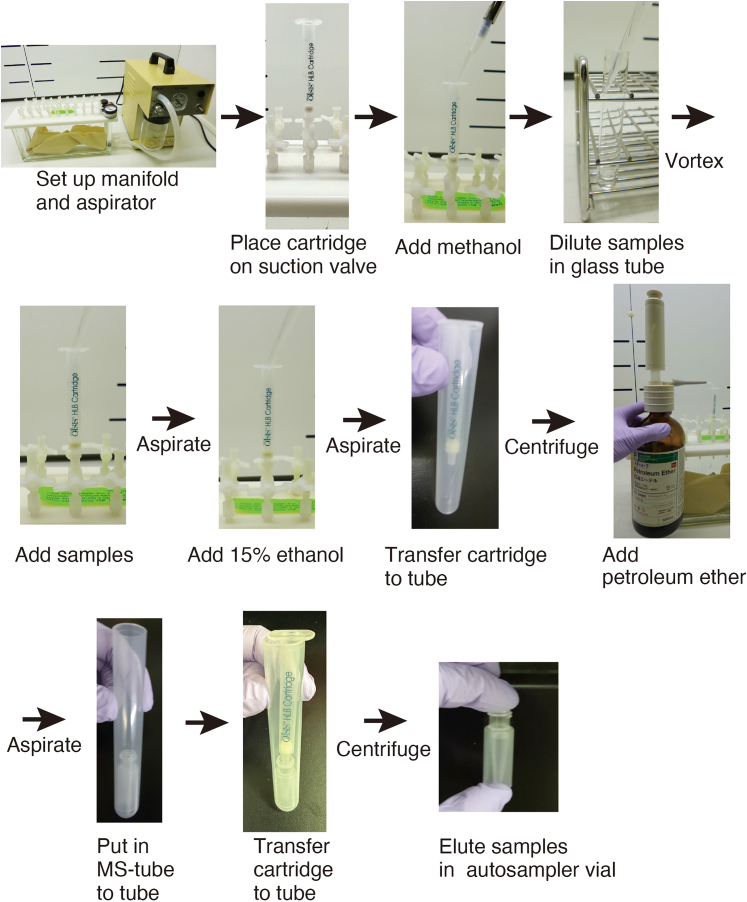
Workflow for purification and enrichment of fatty acid metabolites

This step describes a procedure for the purification and enrichment of fatty acids and their metabolites using a conventional or an improved protocol. An overview of this procedure is shown in Figure 2.

8. Purification Protocol 1: Conventional protocol.
  ***Note:*** This is suitable for tetranor-PGEM, TXB_2_, PGF_2α_, 6-keto-PGF_1α_, PGE_2_, and PGD_2_.
  a. Place an Oasis HLB 1cc (10 mg) extraction cartridge (Waters) on a manifold connected to an aspirator.
  b. Turn on the suction valves, and apply 0.15 mL methanol to the cartridge and wait for 1 min until permeation, then turn off the valves.
  c. Add 1 mL of water:formic acid (100:0.03) to the cartridge, switch on the aspirator, and turn on the valves.
    ***Note:*** Turn off the valve when 0.1–0.2 mL of solution remains in the cartridge.
  d. Dilute the extracted sample with three volumes of water:formic acid (100:0.03) and mix using a vortex mixer. Then, quickly add the diluted samples to the cartridge and turn on the valve.
    ***Note:*** Perform these steps one by one.
  e. Add 1 mL water:formic acid (100:0.03) to the cartridge under aspiration, then turn off the valve.
    ***Note:*** Perform these steps one by one.
  f. Add 1 mL of 15% ethanol in water:formic acid (100:0.03) to the cartridge, then turn on the valve. Once the solvent completely exits the cartridge, turn off the valve and switch off the aspirator.
  g. Transfer the cartridge to an empty adaptor tube, and centrifuge at 240 × *g* for 2 min at 20 to 24°C. with a swing rotor.
  h. Reset the cartridge on the manifold and turn on the valves.
  i. Add 1 mL petroleum ether to the cartridge, and switch on the aspirator. Once the solvent completely exits the cartridge, turn off the valve and switch off the aspirator. (∗At this step, neutral lipids are removed to reduce the ion suppression during mass spectrometry analysis. Tissues such as plasma and liver contain a large amount of neutral lipids.)
  j. Transfer the cartridge to a new adaptor tube fitted with a new autosampler vial (see Figure 2).
    ***Note:*** Ensure the cartridge tip is inserted into the autosampler vial.
  k. Add 0.2 mL of 0.2% formic acid in methanol to the cartridge.
  l. Centrifuge at 240 × *g* for 2 min at 20 to 24°C.
    ***Note:*** If the detection level of the samples is low, the samples should be concentrated up to 10-fold using an evaporator.
9. Purification Protocol 2: Improved protocol.
  ***Note:*** This is suitable for DHA, AA, OEA, EPA, LA, Lyso-PAF, and PAF.
  a. Place an Oasis HLB 1cc (10 mg) extraction cartridge (Waters) on a manifold connected to an aspirator.
  b. Turn on the suction valves, and apply 0.15 mL methanol to the cartridge and wait for 1 min until permeation, then turn off the valves.
  c. Add 1 mL 60% methanol in water:formic acid (100:0.03) to the cartridge, switch on the aspirator, and turn on the valves.
    ***Note:*** Turn off the valve when 0.1–0.2 mL of solution remains in the cartridge.
  d. Dilute the extracted sample with 0.67 volumes of water: formic acid (100:0.03), and mix using a vortex mixer. Then, quickly add the diluted samples to the cartridge and turn on the valve.
    ***Note:*** Perform these steps one by one.
  e. Add 1 mL 60% methanol in water: formic acid (100:0.03) to the cartridge under aspiration, then turn off the valve.
    ***Note:*** Perform these steps one by one.
  f. Add After treatment of all samples, switch off the aspirator.
  g. Transfer the cartridge to an empty adaptor tube, and centrifuge at 240 × *g* for 2 min at 20 to 24°C. with a swing rotor.
  h. Reset the cartridge on the manifold and turn on the valves.
  i. Add 1 mL petroleum ether to the cartridge, and switch on the aspirator. Once the solvent completely exits the cartridge, turn off the valve and switch off the aspirator.
  j. Transfer the cartridge to a new adaptor tube fitted with a new autosampler vial (see Figure 2).
    ***Note:*** Ensure the cartridge tip is inserted into the autosampler vial.
  k. Add 0.2 mL of 0.2% formic acid in methanol to the cartridge.
  l. Centrifuge at 240 × *g* for 2 min at 20 to 24°C.
    ***Note:*** If the detection level of the samples is low, the samples should be concentrated up to 10-fold using an evaporator.

### LC-MS manipulation

**Timing: 30 min/sample**

This step describes how to create an acquisition batch.

10. Preparate the solvents A and B.

***Note:*** Prepare solvent A immediately before use.

11. Place the LC column (Kinetex C8, 2.1 × 150 mm, 2.6 μm, Phenomenex).
12. Purge the two pumps (Conditions: 8 mL/min, 2 min).
13. Switch on LC and MS systems, and equilibrate with the solvents A and B.
14. Load the samples onto the autosampler.
15. Create the batch file (the order list) and download the measurement method.
16. Start a measurement using this batch file. troubleshooting 1.

**Figure 3:**
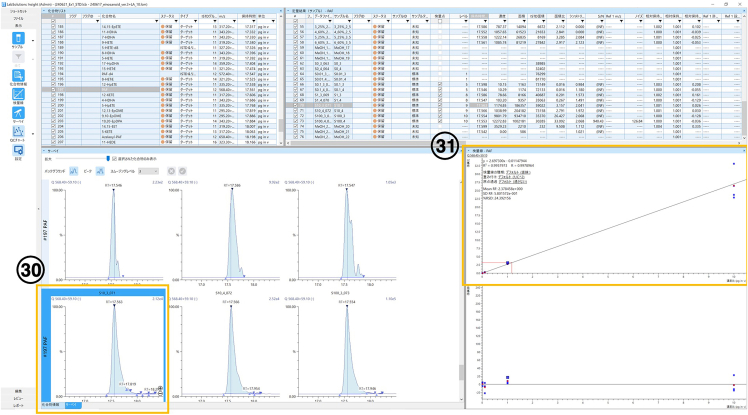
Location of step 30 and 31 on software screen

### Data analysis

**Timing: 6 h**

17. Analyze data using LabSolutions Insight software.
18. Check the peak retention time and peak shape. troubleshooting 2.

∗ Some molecules having the same m/z show multiple peaks. To identify the target molecules, please see Kita *et al.* 2005 and Yamada *et al.* 2015.^3^^,^^4^

19. Check the standard curve (see Figure 3).
20. Export the analyzed data.
21. Divide the value (pg/sample) by tissue weight (mg).

## Expected outcomes

We compared the peak area values and coefficients of variation (CV) of 24 ISTD compounds between the two purification protocols (Table 6). As shown in Table 6, each protocol was suitable for specific molecules. The area ratio (Protocol 2/Protocol 1) depended on the molecules. The CV was improved by an area value increase.

**Table 6:** Area averages and CVs of ISTD compounds (n=3)

Compound	Purification protocol 1		Purification protocol 2		Area ratio
	Area average	CV (%)	Area average	CV (%)	Protocol2/Protocol1
LTD_4_-d5	118033	2.4	43614	7.2	0.37
LTC_4_-d5	117785	7.8	49085	2.5	0.42
LTE_4_-d5	162407	7.5	75466	2.3	0.46
Lyso-PAF-d4	64736	4.9	142022	4.5	2.19
AEA-d4	146260	5.4	197560	3.8	1.35
OEA-d4	224131	9.6	616515	3.0	2.75
tetranor-PGEM-d6	148218	5.0	2935	9.3	0.02
6-keto-PGF_1a_-d4	130111	0.8	20083	5.6	0.15
TXB_2_-d4	393575	1.2	37909	5.0	0.10
PGF_2a_-d9	84177	4.3	8892	5.6	0.11
PGE_2_-d4	426775	0.6	71108	6.1	0.17
PGD_2_-d4	334032	1.5	61408	4.2	0.18
PGA_2_-d4	330891	0.6	131260	5.0	0.40
LTB_4_-d4	134090	1.4	74723	5.7	0.56
14,15-DiHET-d11	328921	1.2	202316	4.7	0.62
15-HETE-d8	126441	2.4	89464	1.0	0.71
12-HETE-d8	92709	1.8	69359	3.7	0.75
5-HETE-d8	34068	4.8	37470	4.6	1.10
PAF-d4	23033	3.8	55429	0.7	2.41
11,12-EET-d11	12276	4.5	10390	7.9	0.85
EPA-d5	23340	14.2	51675	2.5	2.21
DHA-d5	3097	13.2	12053	1.8	3.89
AA-d8	110286	10.6	400992	2.9	3.64
LA-d4	1878122	2.0	4140710	1.9	2.20

Area averages and CVs were calculated from three individual samples.

Orange columns indicate that the area ratio is high (>2.0), so Protocol 2 is suitable for these compounds. Protocol 1 is appropriate for the green-highlighted compounds because the area ratio is low (<0.2).

**Figure 4:**
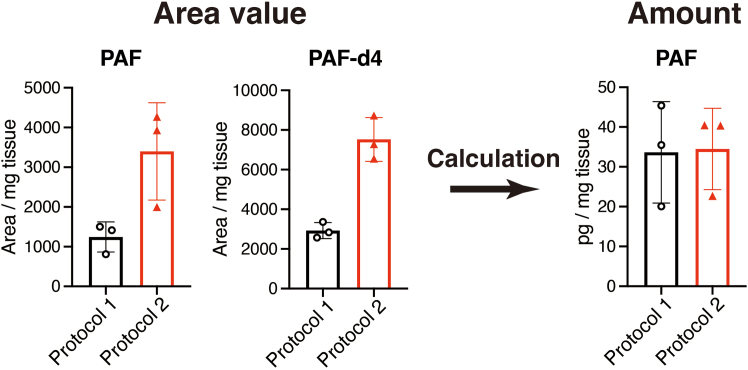
Area values of PAF C16:0 and PAF-d4 and the normalized PAF level in mouse spleen Mean ± SD, n = 3.

Using two purification protocols, we measured four fatty acids and 195 lipid mediators in mouse spleens. The PAF area was higher with the improved protocol (Protocol 2) than with the conventional protocol (Protocol 1) (Figure 4; Table 7). The amount of PAF in 1 mg spleen, calculated using ISTD (PAF-d4), was the same between the two purification protocols (Figure 4). 4-HDHA was detected only using the Protocol 2. However, TXB_2_, PGF_2_α, 8-iso-PGE_2_, PGD_1_, 15-keto-PGE_2_, 13, 14-dihydro-15-keto-PGD_2_, 8-iso-PGA_1_, PGA_2_, PGJ_2_, 17,18-DiHETE, and 17-HDHA were calculated using the Protocol 1. Therefore, the best purification method varied depending on the fatty acid metabolites. The amounts of fatty acid metabolites (pg/mg tissue) were almost the same in both protocols; however, the 15-KEDE, 15-KETE, Azelaoyl-PAF, and 11-HEDE values were different between protocols 1 and 2 because the background may have affected the measurement (data not shown).

**Table 7:** Results of lipid mediators and fatty acids in mouse spleen (n=3)

Compound	Purification protocol 1	Purification protocol 2
	Area/mg tissue (pg/mg tissue)[Mean ± RSD]	Area/mg tissue (pg/mg tissue) [Mean ± RSD]
Lyso-PAF	907857 ± 55080 (676.3 ± 33.8)	1921770 ± 500557 (668.9 ± 31.7)
PC 16:0_2:0	129337 ± 48593 (212.4 ± 82.3)	247241 ± 118069 (186.5 ± 71.5)
AEA	220 ± 28 (0.30.0)	252 ± 28 (0.2 ± 0.0)
15-KEDE	877 ± 63 (0.9 ± 0.1)	1202 ± 232 (0.4 ± 0.0)
OEA	13909 ± 1666 (11.1 ± 1.4)	29641 ± 5610 (8.5 ± 1.4)
6-keto-PGF_1a_	13428 ± 3680 (44.8 ± 11.7)	1793 ± 2931 (40.2 ± 10.9)
6,15-diketo-13,14-dihydro-PGF_1a_	2895 ± 1235 (59.3 ± 24.7)	481 ± 250 (66.2 ± 32.8)
TXB_2_	12712 ± 6668 (33.5 ± 17.5)	ND (ND)
PGF_2a_	1031 ± 549 (5.3 ± 2.7)	ND (ND)
8-iso-PGE_2_	1119 ± 560 (1.8 ± 0.9)	ND (ND)
PGE_2_	27239 ± 13998 (64.0 ± 32.3)	4332 ± 325 (68.6 ± 33.5)
PGD_2_	64323 ± 33128 (142.6 ± 73.6)	10985 ± 4333 (146.6 ± 76.9)
PGD_1_	1163 ± 672 (4.9 ± 2.9)	ND (ND)
15-keto-PGE_2_	374 ± 195 (3.0 ± 1.6)	ND (ND)
13,14-dihydro-15-keto-PGE_2_	914 ± 426 (5.0 ± 2.3)	224 ± 95 (7.4 ± 4.6)
13,14-dihydro-15-keto-PGD_2_	777 ± 424 (2.9 ± 1.6)	ND (ND)
8-iso-PGA_1_	113 ± 15 (0.3 ± 0.0)	ND (ND)
PGA_2_	194 ± 18 (0.5 ± 0.0)	ND (ND)
PGJ_2_	256 ± 151 (1.4 ± 0.8)	ND (ND)
17,18-DiHETE	222 ± 35 (0.7 ± 0.1)	ND (ND)
12,13-DiHOME	6078 ± 1630 (5.2 ± 1.5)	2898 ± 1209 (5.3 ± 1.3)
9,10-DiHOME	1916 ± 382 (2.6 ± 0.6)	1056 ± 404 (3.1 ± 0.9)
19,20-DiHDPA	246 ± 34 (7.6 ± 1.1)	171 ± 68 (5.1 ± 1.1)
12-HHT	533 ± 287 (30.0 ± 16.1)	290 ± 131 (34.3 ± 16.3)
15-HEPE	208 ± 69 (2.3 ± 0.8)	172 ± 92 (2.3 ± 1.0)
12-HEPE	832 ± 344 (7.4 ± 3.1)	684 ± 281 (7.1 ± 2.9)
13-HODE	7027 ± 2223 (10.5 ± 3.3)	5719 ± 1471 (10.0 ± 2.8)
9-HODE	4489 ± 1948 (44.5 ± 19.2)	3891 ± 1667 (44.8 ± 19.2)
15-HETE	2613 ± 994 (13.8 ± 5.2)	2184 ± 759 (13.5 ± 4.6)
13-KODE	335 ± 65 (1.6 ± 0.3)	378 ± 54 (2.1 ± 0.2)
17-HDHA	220 ± 67 (4.7 ± 1.5)	ND (ND)
11-HETE	10313 ± 4193 (19.6 ± 8.1)	9193 ± 3684 (18.6 ± 7.6)
13-HDHA	466 ± 39 (1.4 ± 0.1)	480 ± 164 (1.5 ± 0.5)
14-HDHA	790 ± 171 (16.03.6)	665 ± 170 (14.3 ± 2.4)
12-HETE	10653 ± 4135 (57.8 ± 22.9)	9728 ± 3996 (55.8 ± 22.4)
15-KETE	355 ± 87 (4.7 ± 1.5)	389 ± 80 (1.8 ± 0.1)
15-HETrE	598 ± 295 (2.7 ± 1.4)	558 ± 310 (2.3 ± 1.2)
PAF	1243 ± 308 (33.7 ± 10.4)	3400 ± 1557 (34.5 ± 8.3)
4-HDHA	ND (ND)	131 ± 31 (1.2 ± 0.0)
Azelaoyl-PAF	2816 ± 545 (24.2 ± 8.1)	3361 ± 858 (10.8 ± 2.4)
11-HEDE	594 ± 265 (17.1 ± 8.7)	579 ± 271 (3.3 ± 1.6)
EPA	539 ± 92 (178.3 ± 34.1)	1444 ± 598 (177.1 ± 41.5)
DHA	8289 ± 384 (753.1 ± 107.8)	42887 ± 1864 (724.2 ± 94.7)
AA	42394 ± 13248 (640.1 ± 207.5)	228165 ± 124229 (694.5 ± 237.2)

The averages and relative standard deviations (RSDs) were calculated using three independent samples. ND indicates “not detected.” Light yellow columns indicate the higher area value between the purification protocols 1 and 2.

Next, we measured the metabolites in the brain (Table 8). In the brain, 59 compounds were detected using the protocols 1 and 2, respectively. Among these, five compounds were detected only in the Protocol 1, and other five compounds were detected in the Protocol 2. The calculated values of DHA and AA were different between protocols 1 and 2 in the brain but not in the spleen. These results suggested that endogenous DHA and AA may suppress the ionization of the internal standard DHA-d5 and AA-d4 because the amounts of endogenous DHA and AA in the brain was higher than that in the spleen. Therefore, the DHA and AA values in the brain may not be accurately quantified by either protocol. Similar to the spleen, TXB_2_, PGD_3_, 8-iso-PGA_1_, 18-HEPE, and 13-HODE in the brain were only detected using the Protocol 1. AEA, PGD_1_, and 13,14-dihydro-15-keto-PGD_2_ were measured using both methods in the brain but were only calculated using the Protocol 1 in the spleen. They can be detected using both methods in the brain because the weight of brain used in the protocols was higher than that of the spleen. Therefore, this method should be selected based on the compound class and tissue amount.

**Table 8:** Results of lipid mediators and fatty acids in mouse brain (n=3)

Compound	Purification protocol 1	Purification protocol 2
	Area/mg tissue (pg/mg tissue) [mean ± RSD]	Area/mg tissue (pg/mg tissue) [mean ± RSD]
Lyso-PAF	30428 ± 2140 (48.4 ± 1.6)	115061 ± 6889 (52.0 ± 3.5)
PC 16:0_2:0	9472 ± 693 (32.8 ± 3.0)	30454 ± 636 (30.0 ± 2.6)
AEA	563 ± 26 (1.2 ± 0.2)	1345 ± 283 (1.3 ± 0.2)
15-KEDE	57 ± 4 (0.2 ± 0.0)	115 ± 7 (0.1 ± 0.0)
OEA	10007 ± 583 (26.7 ± 1.9)	68859 ± 13070 (29.2 ± 2.4)
6-keto-PGF_1a_	1546 ± 209 (4.8 ± 0.8)	157 ± 22 (3.6 ± 0.7)
8-iso-15(R)-PGF_2a_	203 ± 15 (1.0 ± 0.1)	12 ± 2 (0.7 ± 0.1)
TXB_2_	5924 ± 290 (15.5 ± 1.3)	ND (ND)
PGD_3_	67 ± 9 (0.3 ± 0.0)	ND (ND)
PGF_2a_	4320 ± 336 (22.3 ± 2.2)	414 ± 47 (25.4 ± 2.5)
8-iso-PGE_2_	365 ± 7 (0.5 ± 0.0)	57 ± 16 (0.7 ± 0.2)
PGE_2_	3781 ± 400 (8.6 ± 1.2)	521 ± 67 (9.2 ± 1.4)
15-keto-PGF_2a_	49 ± 7 (0.4 ± 0.1)	8 ± 2 (0.5 ± 0.1)
PGE_1_	563 ± 51 (2.3 ± 0.3)	85 ± 13 (2.4 ± 0.3)
PGD_2_	51057 ± 4908 (111.7 ± 14.8)	7478 ± 1021 (112.7 ± 14.6)
PGD_1_	128 ± 19 (0.5 ± 0.1)	19 ± 3 (0.5 ± 0.1)
13,14-dihydro-15-keto-PGD_2_	101 ± 14 (0.4 ± 0.1)	32 ± 5 (0.8 ± 0.1)
8-iso-PGA_1_	11 ± 2 (0.0 ± 0.0)	ND (ND)
PGJ_2_	291 ± 40 (1.7 ± 0.3)	78 ± 13 (0.9 ± 0.1)
8,15-DiHETE	29 ± 0 (0.5 ± 0.0)	11 ± 3 (0.3 ± 0.1)
17,18-DiHETE	60 ± 17 (0.2 ± 0.0)	22 ± 5 (0.1 ± 0.0)
5,15-DiHETE	167 ± 3 (1.0 ± 0.0)	66 ± 6 (0.7 ± 0.0)
12,13-DiHOME	538 ± 141 (0.5 ± 0.1)	227 ± 59 (0.4 ± 0.1)
9,10-DiHOME	161 ± 29 (0.2 ± 0.0)	77 ± 13 (0.2 ± 0.0)
19,20-DiHDPA	34 ± 9 (2.0 ± 0.3)	24 ± 1 (0.8 ± 0.0)
14,15-DHET	59 ± 3 (0.1 ± 0.0)	40 ± 4 (0.2 ± 0.0)
12-HHT	434 ± 17 (26.3 ± 2.3)	270 ± 35 (33.5 ± 4.1)
11,12-DHET	20 ± 2 (0.1 ± 0.0)	12 ± 1 (0.1 ± 0.0)
18-HEPE	10 ± 2 (0.2 ± 0.0)	ND (ND)
16-HETE	125 ± 7 (0.9 ± 0.1)	91 ± 2 (0.8 ± 0.0)
13-HODE	266 ± 23 (0.4 ± 0.0)	ND (ND)
9-HODE	100 ± 5 (1.0 ± 0.1)	81 ± 2 (1.0 ± 0.0)
20-HDHA	131 ± 8 (1.5 ± 0.1)	139 ± 13 (1.9 ± 0.2)
15-HETE	3366 ± 129 (18.9 ± 1.1)	2997 ± 304 (19.8 ± 1.7)
9-HpODE	20 ± 2 (0.4 ± 0.0)	34 ± 4 (0.8 ± 0.1)
13-KODE	50 ± 11 (0.3 ± 0.1)	50 ± 7 (0.3 ± 0.0)
13-HpODE	20 ± 5 (0.3 ± 0.1)	31 ± 4 (0.5 ± 0.1)
16-HDHA	122 ± 8 (0.8 ± 0.1)	129 ± 16 (0.8 ± 0.1)
17-HDHA	44 ± 13 (1.2 ± 0.3)	46 ± 9 (1.2 ± 0.2)
11-HETE	6291 ± 253 (15.0 ± 1.0)	6529 ± 872 (14.8 ± 1.9)
13-HDHA	120 ± 10 (0.4 ± 0.1)	133 ± 11 (0.5 ± 0.0)
10-HDHA	33 ± 5 (0.5 ± 0.1)	46 ± 7 (0.7 ± 0.1)
8-HETE	394 ± 23 (3.9 ± 0.3)	452 ± 45 (4.3 ± 0.5)
14-HDHA	30 ± 13 (0.7 ± 0.3)	37 ± 4 (0.9 ± 0.1)
12-HETE	1092 ± 62 (7.4 ± 0.4)	1224 ± 68 (7.9 ± 0.5)
15-KETE	1557 ± 48 (67.6 ± 6.6)	2820 ± 308 (19.7 ± 2.0)
15-HpETE	1065 ± 56 (27.7 ± 0.7)	2774 ± 310 (68.5 ± 5.5)
11-HDHA	19 ± 2 (0.5 ± 0.1)	29 ± 3 (0.4 ± 0.0)
9-HETE	103 ± 9 (5.4 ± 0.6)	142 ± 17 (4.2 ± 0.5)
8-HDHA	ND (ND)	27 ± 1 (0.9 ± 0.1)
5-HETE	330 ± 5 (5.3 ± 0.4)	609 ± 61 (5.6 ± 0.4)
17-HpDHA	ND (ND)	24 ± 5 (1.3 ± 0.3)
15-HETrE	78 ± 10 (0.7 ± 0.1)	77 ± 9 (0.4 ± 0.0)
12-HpETE	187 ± 23 (18.0 ± 0.8)	490 ± 49 (27.3 ± 2.1)
PAF	10 ± 3 (0.8 ± 0.2)	57 ± 5 (1.1 ± 0.0)
4-HDHA	26 ± 2 (0.5 ± 0.1)	71 ± 6 (0.8 ± 0.0)
5-HpETE	23 ± 2 (1.7 ± 0.2)	132 ± 27 (5.7 ± 1.1)
5-KETE	189 ± 21 (15.1 ± 0.4)	438 ± 35 (5.7 ± 0.5)
Azelaoyl-PAF	ND (ND)	20 ± 5 (0.1 ± 0.0)
11-HEDE	ND (ND)	12 ± 1 (0.1 ± 0.0)
5,6-EET	ND (ND)	50 ± 13 (2.3 ± 0.9)
EPA	278 ± 11 (254.0 ± 34.7)	1816 ± 384 (258.6 ± 34.9)
DHA	12775 ± 478 (4840.6 ± 571.2)	145720 ± 21532 (3810.2 ± 230.1)
AA	227507 ± 11669 (6838.4 ± 494.2)	1360374 ± 57847 (4000.3 ± 363.9)

The average amounts and RSDs were calculated using three independent samples. ND indicates “not detected.”

Finally, these parameters were measured in the lung (Table 9). Using protocols 1 and 2, 51 and 55 compounds were detected, respectively. Among these, eight compounds were detected in the Protocol 1 and 11 compounds were detected in the Protocol 2. Similar to the spleen, PGD_1_ and 13,14-dihydro-15-keto-PGD_2_ were only detected using the Protocol 1. AEA was detected using the both protocols in the lung as well as in the brain.

**Table 9:** Results of lipid mediators and fatty acids in mouse lungs (n=3)

Compound	Purification protocol 1	Purification protocol 2
	Area/mg tissue (pg/mg tissue) [mean ± RSD]	Area/mg tissue (pg/mg tissue) [mean ± RSD]
Lyso-PAF	236779 ± 22985 (529.9 ± 51.1)	1176751 ± 60657 (514.4 ± 64.2)
PC 16:0_2:0	16189 ± 7695 (78.3 ± 38.5)	64923 ± 25282 (63.5 ± 31.1)
AEA	72 ± 33 (0.2 ± 0.1)	275 ± 85 (0.3 ± 0.1)
15-KEDE	154 ± 32 (0.6 ± 0.2)	412 ± 114 (0.2 ± 0.1)
OEA	6734 ± 3881 (20.6 ± 9.3)	56660 ± 29349 (19.8 ± 9.0)
6-keto-PGF_1a_	31117 ± 20026 (130.4 ± 86.7)	5187 ± 3374 (130.2 ± 89.1)
6,15-diketo-13,14-dihydro-PGF_1a_	3840 ± 2475 (98.9 ± 65.9)	1003 ± 703 (155.5 ± 113.8)
8-iso-15(R)-PGF_2a_	236 ± 155 (1.5 ± 1.0)	ND (ND)
PGE_3_	427 ± 246 (1.9 ± 1.2)	ND (ND)
PGF_2a_	2403 ± 1432 (15.7 ± 9.7)	295 ± 194 (15.7 ± 10.0)
PGF_1a_	347 ± 232 (3.4 ± 2.3)	ND (ND)
8-iso-PGE_2_	1238 ± 805 (2.3 ± 1.6)	235 ± 158 (2.5 ± 1.7)
PGE_2_	28490 ± 17058 (80.5 ± 50.8)	4979 ± 3163 (80.0 ± 51.7)
8-iso-PGE_1_	87 ± 62 (0.7 ± 0.5)	9 ± 7 (0.4 ± 0.3)
15-keto-PGF_2a_	1304 ± 1065 (12.9 ± 10.9)	295 ± 260 (16.4 ± 14.6)
PGE_1_	2587 ± 1654 (13.1 ± 8.7)	509 ± 349 (13.7 ± 9.4)
PGD	12995 ± 8975 (34.8 ± 25.0)	2459 ± 1784 (34.9 ± 25.4)
PGD_1_	931 ± 666 (4.8 ± 3.5)	ND (ND)
15-keto-PGF_1a_	293 ± 253 (0.9 ± 0.8)	ND (ND)
15-keto-PGE_2_	6416 ± 4846 (63.7 ± 50.3)	1616 ± 1251 (84.9 ± 65.9)
13,14-dihydro-15-keto-PGF_2a_	1699 ± 1186 (20.7 ± 15.0)	ND (ND)
13,14-dihydro-15-keto-PGE_2_	13898 ± 9554 (91.1 ± 64.9)	4122 ± 2929 (143.5 ± 102.4)
1a,1b-dihomo-PGF_2_	374 ± 232 (0.9 ± 0.6)	ND (ND)
13,14-dihydro-15-keto-PGD_2_	531 ± 329 (2.4 ± 1.6)	199 ± 142 (4.8 ± 3.4)
PGA_2_	87 ± 36 (0.2 ± 0.1)	ND (ND)
17,18-DiHETE	238 ± 48 (0.9 ± 0.2)	128 ± 41 (0.7 ± 0.2)
12,13-DiHOME	5449 ± 1667 (6.2 ± 1.5)	2785 ± 851 (5.9 ± 1.8)
9,10-DiHOME	1830 ± 547 (3.3 ± 0.8)	1055 ± 348 (3.6 ± 1.2)
19,20-DiHDPA	277 ± 86 (24.2 ± 9.0)	222 ± 68 (8.7 ± 3.0)
14,15-DHET	143 ± 42 (0.4 ± 0.1)	73 ± 30 (0.4 ± 0.2)
12-HHT	369 ± 242 (28.2 ± 19.7)	246 ± 159 (33.7 ± 21.7)
20-carboxy-AA	ND (ND)	41 ± 5 (3.8 ± 0.5)
13-HOTrE	101 ± 61 (2.7 ± 1.5)	87 ± 46 (4.4 ± 2.3)
15-HEPE	243 ± 108 (3.8 ± 1.6)	255 ± 152 (3.7 ± 2.1)
11-HEPE	124 ± 63 (46.3 ± 24.7)	115 ± 63 (8.5 ± 5.0)
12-HEPE	1109 ± 721 (14.2 ± 9.5)	1081 ± 679 (12.9 ± 8.3)
13-HODE	8361 ± 4435 (17.9 ± 9.6)	6960 ± 3636 (13.8 ± 7.4)
9-HODE	5492 ± 3250 (77.7 ± 46.3)	4777 ± 2889 (63.3 ± 39.6)
15-HETE	2451 ± 1530 (18.9 ± 12.6)	2768 ± 1732 (19.8 ± 13.2)
13-KODE	181 ± 70 (1.2 ± 0.5)	318 ± 122 (2.0 ± 0.7)
13-HpODE	ND (ND)	112 ± 41 (2.0 ± 0.8)
16-HDHA	154 ± 82 (1.2 ± 0.7)	148 ± 54 (0.9 ± 0.4)
17-HDHA	362 ± 254 (12.2 ± 7.9)	491 ± 280 (13.3 ± 7.3)
9-KODE	ND (ND)	63 ± 17 (0.8 ± 0.2)
11-HETE	8929 ± 5595 (27.6 ± 18.4)	11966 ± 7589 (29.1 ± 19.2)
13-HDHA	619 ± 373 (2.9 ± 1.8)	902 ± 549 (3.3 ± 2.0)
10-HDHA	ND (ND)	202 ± 124 (3.0 ± 1.7)
8-HETE	ND (ND)	79 ± 36 (0.8 ± 0.4)
14-HDHA	1137 ± 725 (36.1 ± 21.4)	1695 ± 982 (43.0 ± 24.2)
12-HETE	8536 ± 5670 (75.2 ± 52.5)	11504 ± 7824 (79.5 ± 55.7)
15-KETE	ND (ND)	255 ± 114 (1.5 ± 0.7)
8-HDHA	33 ± 14 (2.8 ± 1.2)	79 ± 12 (3.0 ± 0.3)
5-HETE	ND (ND)	92 ± 34 (1.0 ± 0.3)
15-HETrE	1458 ± 949 (19.3 ± 14.2)	1916 ± 1223 (11.3 ± 7.7)
PAF	ND (ND)	180 ± 39 (3.0 ± 0.8)
4-HDHA	ND (ND)	129 ± 52 (1.6 ± 0.7)
Azelaoyl-PAF	127 ± 14 (3.4 ± 0.4)	287 ± 61 (1.5 ± 0.2)
11-HEDE	ND (ND)	830 ± 540 (6.3 ± 4.5)
15-HEDE	ND (ND)	137 ± 74 (0.9 ± 0.5)
EPA	191 ± 54 (238.2 ± 48.1)	1681 ± 439 (224.9 ± 57.4)
DHA	4133 ± 1422 (2271.4 ± 556.1)	70210 ± 25308 (1532.8 ± 546.9)
AA	7888 ± 1238 (383.1 ± 55.0)	121974 ± 14636 (472.4 ± 110.1)

The average amounts and RSDs were calculated using three independent samples. ND indicates “not detected.”

## Quantification and statistical analysis

Compounds were calculated using the calibration curves of their respective standards.

## Limitations

We investigated a purification method for analyzing many lipid mediators simultaneously. Although we did not examine precise sample condition (such as tissue type, tissue weight, and column size) in this study, the number of detected compounds depended on the sample conditions. The signals detected by LC–MS can be affected by the sample matrix.

Please see our literatures using the Protocol1^6^^,^^7^^,^^8^^,^^9^ and the Protocol 2.^1^^,^^10^^,^^11^^,^^12^^,^^13^

## Troubleshooting

### Problem 1

LC pressure increases after running several samples (Step 26).

### Potential solution

Wash the LC line and the column with isopropanol to avoid any accumulation.

### Problem 2

Retention times of metabolites are shift compared to previous measurements.

### Potential solution

Measure the standard (STD) at each time and match the elution times of metabolites.

## Resource availability

### Lead contact

Further information and requests for resources and reagents should be directed to and will be fulfilled by the lead contact, Hidao Shindou (shindou.h@jihs.go.jp).

### Technical contact

Technical questions on executing this protocol should be directed to and will be answered by the technical contacts, Tomomi Hashidate-Yoshida (hashidate.t@jihs.go.jp), Masaki Yamada (yamada@shimadzu.co.jp), and Yoshihiro Kita (kita@m.u-tokyo.ac.jp).

### Materials availability

This study did not generate new unique materials.

### Data and code availability

The datasets and MS instrument file supporting the current study have not been deposited in a public repository because of the company’s confidentiality policy but are available from the corresponding author upon reasonable request.

## Acknowledgments

We are grateful for the constructive comments from Prof. Takao Shimizu and all the members of our laboratory (JIHS). We thank Mio Yamada, Yukiko Sugimoto, and Chika Furumoto (JIHS) for their technical support. We would like to thank Editage (www.editage.jp) for English-language editing.

This work was supported by the 10.13039/100009619AMED Program for Basic and Clinical Research on Hepatitis (grant no. 23fk0210091 to H.S.), the Japan Institute for Health Security (JIHS) Intramural Research Fund (22T001, 24A1013, and 24A2011 to H.S.), the 10.13039/100020237Japan Health Research Promotion Bureau Research Fund (JH2022-B-03 to H.S.), and 10.13039/501100008664ONO Medical Research Foundation (to H.S.). T.H.-Y. was supported by 10.13039/501100001691MEXT KAKENHI (grant no. JP23738333).

## Author contributions

T.H.-Y. designed the study, performed the experiments, analyzed the data, and wrote the manuscript. M.Y. analyzed the data and wrote the manuscript. K.N. performed the experiments and analyzed the data. Y. Kihara and Y. Kita edited the manuscript. H.S. designed and drafted the manuscript. All authors assisted in editing the manuscript.

## Declaration of interests

The Department of Lipid Life Science (Japan Institute for Health Security, JIHS) collaborated with the Shimadzu Corporation. One of the authors is a researcher at the Shimadzu Corporation.

## Supplemental Information

Table S1. Information of ISTD and STD, related to Before You Begin Step 1
